# The Effect of a Novel Natural Oil-Pulling Oral Rinse on Oral Microflora, Dental Plaque, and Gingivitis: A Double-Blinded Randomized Controlled Clinical Trial

**DOI:** 10.7759/cureus.113440

**Published:** 2026-07-27

**Authors:** Chetan Patel, Ghanashyam Patel, Nile Desai

**Affiliations:** 1 Pharmaceutical Sciences, University of Greenwich, Chatham Maritime, GBR; 2 Integrative Medicine, Gujarat Ayurved University, Jamnagar, IND; 3 Dentistry, Care N Cure Dental Clinic, Valsad, IND

**Keywords:** complementary therapies, dental plaque, essential oils, gingivitis, halitosis, mouthwashes, oil pulling, oral hygiene, oral microbiome, randomized controlled trial

## Abstract

Aim: Oral health conditions such as plaque accumulation, gingivitis, and halitosis are primarily caused by pathogenic oral microflora and remain a global concern due to their links with systemic diseases. While essential oil-based mouthwashes are effective, concerns over side effects such as dryness and taste alterations have spurred interest in natural alternatives such as oil-pulling rinses. This randomized, double-blind, placebo-controlled clinical trial evaluated the efficacy and safety of a novel oil-pulling oral rinse compared to an alcohol-based anti-gingivitis mouthwash and placebo in reducing oral microbial counts, dental plaque, and gingivitis.

Materials and methods: A total of 63 adults (aged 18-50 years) with a modified gingival index (MGI) ≥ 1.75 and plaque index (PI) ≥ 1.95 were randomized into three groups: an oil-pulling oral rinse (test group), a marketed alcohol-based anti-gingivitis mouthwash (active comparator), and placebo. Participants used their assigned mouth rinse for 15 days. Primary outcomes included changes in oral microflora (colony-forming unit (CFU) counts of gram-positive and gram-negative bacteria and *Candida albicans*), MGI, and PI. Secondary outcomes assessed oral dryness (Clinical Oral Dryness Score (CODS)), breath freshness (Organoleptic Scoring Scale (OSS)), and tooth shade (VITA Shade Scale; VITA Zahnfabrik, Bad Säckingen, Germany). Adverse events (AEs) and safety parameters were monitored.

Results: The test group demonstrated a numerically greater reduction in gram-positive bacteria (69.3%) compared with the comparator (63.5%) and placebo (51.6%); however, these differences were not statistically significant. Gram-negative bacterial reduction was significantly greater in the test group (24.9%) than in the active comparator (22.1%) and placebo (11.0%) groups (p = 0.004). MGI improved by 2.7% (p = 0.015), and PI was reduced by 30.6% (p < 0.001). The test group also showed improvements in oral dryness (p < 0.001), breath freshness (p < 0.001), and teeth shade (p = 0.011). No serious AEs were reported.

Conclusion: The evaluated oil-pulling oral rinse showed potential to reduce oral microbial counts and improve plaque and gingival parameters during short-term use. It presents a natural, well-tolerated alternative to conventional mouthwashes.

## Introduction

Oral hygiene plays a pivotal role in maintaining overall health and preventing systemic diseases. The accumulation of dental plaque and the proliferation of oral microflora are primary factors contributing to oral health conditions such as gingivitis, periodontitis, and malodor or halitosis [1]. These conditions, if left untreated, can lead to systemic complications, including cardiovascular diseases, diabetes, and respiratory infections [2-4]. Consequently, the development and evaluation of effective oral care products remain crucial for advancing preventive healthcare strategies.

Mouth rinses have been extensively used as adjuncts to mechanical oral hygiene practices such as brushing and flossing. Among the various products available, essential oil-based mouth rinses and chemical formulations have garnered widespread acceptance for their antimicrobial properties. Oral rinses containing essential oils such as thymol, eucalyptol, and menthol have demonstrated significant efficacy in reducing plaque and gingivitis [5,6]. However, concerns about their potential side effects, including oral dryness and taste alterations, underscore the need for alternative formulations [7].

Oil pulling, a traditional oral hygiene practice rooted in Ayurveda, involves swishing oil in the mouth to improve oral health. Recent formulations, such as the one evaluated in this study, build upon the traditional practice of oil pulling by incorporating blends of functional oils supported by emerging scientific insights. This formulation includes oils traditionally used for oil pulling along with essential oils known for their potential antimicrobial, anti-inflammatory, and soothing properties. Although preliminary studies and anecdotal evidence suggest potential benefits of oil pulling and essential oil-based oral care, robust, well-designed clinical trials evaluating their efficacy and safety remain limited.

Randomized controlled trials (RCTs) serve as the gold standard for evaluating the effectiveness of interventions. Previous studies have demonstrated that essential oil-based mouthwashes containing alcohol are effective in reducing oral microbial counts, plaque accumulation, and gingival inflammation [8-11]. Similarly, essential oils have shown promise in enhancing oral hygiene by exerting antimicrobial and anti-inflammatory effects [12]. However, the efficacy of these products in improving additional parameters such as oral aesthetics, malodor, and dryness has not been comprehensively evaluated in a single trial. Furthermore, there is limited clinical evidence supporting the effectiveness of all-natural, alcohol-free mouthwashes in maintaining overall oral hygiene.

This study aims to address these gaps by comparing the safety and efficacy of the test oral rinse with those of a marketed alcohol-based anti-gingivitis mouthwash and placebo in adult patients requiring oral hygiene support. The primary objectives are to evaluate their impact on oral microflora, dental plaque, and gingivitis. The secondary objectives are to assess their effects on oral malodor, dryness, and teeth brightness, alongside safety and tolerability.

The randomized, double-blind, active-, and placebo-controlled study design ensures methodological rigor, providing high-quality evidence for clinical decision-making. The findings from this trial are expected to advance the understanding of therapeutic oil-based products in oral care and offer valuable insights into their comparative effectiveness against established chemical formulations. By bridging the evidence gap, this research holds the potential to inform clinical practice and guide consumers toward more effective and tolerable oral hygiene solutions.

## Materials and methods

Study design

**Figure 1:**
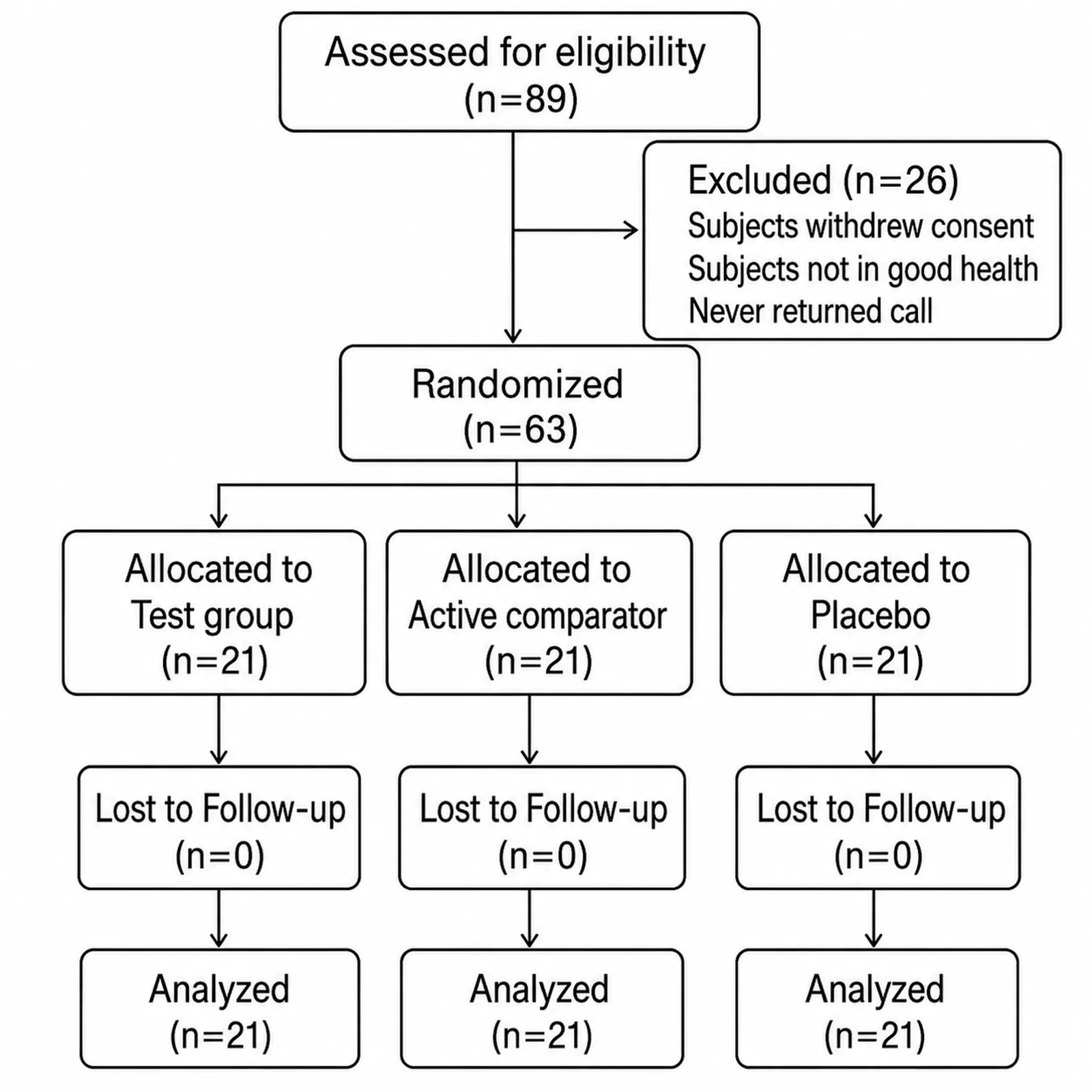
CONSORT diagram CONSORT: Consolidated Standards of Reporting Trials.

This was a randomized, double-blind, active-controlled, placebo-controlled, parallel-group clinical trial. The study followed the CONSORT (Consolidated Standards of Reporting Trials) 2010 guidelines (Figure 1).

Materials

The test product was GuruNanda® Oil Pulling Oral Rinse (GuruNanda LLC, Buena Park, CA), formulated primarily with fractionated coconut oil (medium-chain triglyceride oil) as the base component. The formulation also contained essential oils including peppermint oil, spearmint oil, clove oil, cardamom oil, tea tree oil, oregano oil, and fennel oil, along with stevia leaf extract and vitamins E, D3, and K2.

The essential oils were present in low concentrations typical of oral care formulations (generally <1% individually), whereas fractionated coconut oil constituted the primary carrier base. These components were selected based on their reported antimicrobial, anti-inflammatory, and breath-freshening properties described in prior oral hygiene studies.

Methods

Patient Selection

This study was conducted from July 14, 2023, to August 31, 2023. The study was registered with Clinical Trials Registry - India (Registration number: CTRI/2023/06/054517 dated June 28, 2023). Participants requiring oral hygiene support for controlling pathologic microflora in the oral cavity were identified. Eligible participants fulfilled all inclusion criteria and met none of the exclusion criteria.

Inclusion and Exclusion Criteria

The study population included adults aged 18-50 years with good periodontal health who required oral hygiene support for their gums and teeth to control the pathogenic microflora of the oral cavity, had a modified gingival index (MGI) score ≥ 1.75, and a plaque index (PI) score ≥ 1.95. Participants agreed to use the assigned investigational product (IP), including the provided toothpaste and toothbrush, and to follow the study protocol, including maintaining a 12-hour overnight gap before providing salivary samples.

Exclusion criteria comprised hypersensitivity to oral hygiene products, recent use of antibiotics or specific oral care products, orthodontic treatment, fixed appliances, or tobacco use. Additionally, pregnant or lactating women, individuals with COVID-19 symptoms or severe dental conditions, and participants with a history of addiction to recreational drugs, drug dependence, or alcohol addiction were excluded from the study.

Sample Size Calculation

The sample size was determined based on the primary clinical endpoint of reduction in PI. Previous RCTs evaluating herbal or essential oil mouth rinses have reported plaque reduction differences ranging between 20% and 30% compared with placebo. Assuming a moderate effect size (Cohen’s d ≈ 0.7), a statistical power of 80%, and a two-sided alpha level of 0.05, a minimum of 18 participants per group was required to detect clinically meaningful differences between groups. To account for potential dropouts and incomplete data, the sample size was increased to 21 participants per arm, resulting in a total study population of 63 participants. This sample size is consistent with previously published clinical trials evaluating mouth-rinse interventions in gingivitis and plaque control.

Randomization and Allocation Concealment

Participants were randomly allocated in a 1:1:1 ratio to the test oral rinse, active comparator mouthwash, or placebo group using a computer-generated randomization sequence. The randomization schedule was generated by an independent statistician who was not involved in participant recruitment, clinical assessment, or data analysis.

Allocation concealment was ensured through the use of sequentially numbered, identical product kits prepared by an independent third party. Each kit contained the assigned IP along with its corresponding placebo, where applicable. The kits were labeled with unique participant identification codes according to the randomization schedule.

Study investigators responsible for participant enrollment assigned the next available sequential kit number without knowledge of the treatment allocation. This procedure ensured that group assignment remained concealed from investigators, participants, and outcome assessors throughout the study period.

Blinding

The study was conducted using a double-blind design in which participants, clinical investigators responsible for participant enrollment and follow-up, outcome assessors, microbiology laboratory personnel, and the statistician performing the data analysis were unaware of treatment allocation. The IPs (test oral rinse, active comparator mouthwash, and placebo formulations) were packaged in identical containers with similar labeling, color, and flavor profiles to maintain blinding.

Clinical assessments, including MGI [13], PI [14], Clinical Oral Dryness Score (CODS) [15], Organoleptic Scoring Scale (OSS) [16], and VITA shade evaluation (VITA Zahnfabrik, Bad Säckingen, Germany), were performed by examiners who were blinded to the treatment assignment.

Microbiological analyses of salivary samples were conducted by laboratory personnel who were not involved in clinical assessments and were blinded to group allocation. The statistical analysis was performed using anonymized group codes to ensure that the data analyst remained blinded to the treatment allocation until completion of the primary analysis.

The placebo formulations were designed to closely match the active products in color, flavor, and packaging to maintain effective masking throughout the study.

Study Methodology

Eligible participants underwent screening (Visit 1) approximately seven days before baseline, which served as a run-in period to standardize oral hygiene practices before initiation of the IPs. During this period, participants were instructed to maintain routine oral hygiene practices and avoid the use of additional mouth rinses, antimicrobial oral products, or antibiotics that could influence oral microbial composition. At baseline (Visit 2, Day 0), participants were randomized to receive either the test oral rinse, the active comparator mouthwash, or placebo according to a computer-generated randomization schedule. Details of the test and reference product are listed in Table 1. The assigned products were provided for a 15-day usage period along with a patient diary card (PDC) for compliance documentation. Participants received detailed instructions on IP usage and PDC maintenance.

**Table 1:** Details of test and comparator products used in the study *The active comparator consisted of a commercially available alcohol-based essential oil mouthwash widely used for plaque and gingivitis control. The product contains antimicrobial essential oils in a hydroalcoholic base consistent with commonly marketed anti-gingivitis rinses. **The placebo formulations were designed to mimic the appearance, viscosity, and flavor of the corresponding active products but did not contain antimicrobial essential oils or active botanical components. Placebo preparations included an inert base solution with flavoring agents and colorants to maintain blinding of participants and investigators.

Product detail	Test product	Active comparator^*^	Placebo test product^**^	Placebo active comparator
Formulation	Oil-pulling oral rinse	Commercially available essential oil–based alcohol-containing mouthwash (standard anti-gingivitis formulation)	Placebo	Placebo
Dosage form	Oil pulling	Liquid	Liquid	Liquid
Route of administration	Oral	Oral	Oral	Oral
Manufacturer	GuruNanda LLC	Confidential	Green Medic Solutions	Green Medic Solutions
Batch number	01	01	01	01
Manufacturing date	June 2023	June 2023	June 2023	June 2023
Expiry date	May 2025	May 2025	May 2025	May 2025

Clinical Assessments

Clinical periodontal assessments, including the MGI, PI, CODS, OSS, and VITA shade assessment, were performed by a trained dental examiner experienced in periodontal evaluation. Prior to study initiation, the examiner underwent calibration exercises using volunteer subjects who were not included in the study to ensure consistency in all clinical outcome assessments. Calibration was conducted by repeated examination of the same volunteers after a 24-hour interval under identical clinical conditions.

Intra-examiner reliability was assessed by repeating measurements after a 24-hour interval under identical clinical conditions. Agreement between repeated measurements was evaluated using intra-class correlation coefficients (ICC), with values exceeding 0.80 considered acceptable for clinical reliability. This calibration process ensured standardized and reproducible scoring of periodontal indices throughout the study.

Gingival inflammation was evaluated using the MGI, a noninvasive clinical index that assesses visual signs of gingival inflammation without periodontal probing. Plaque accumulation was assessed using the PI, which evaluates plaque deposition on selected tooth surfaces based on standardized clinical criteria. These indices were recorded at screening (Visit 1), baseline (Visit 2), and the end-of-treatment (EOT) visit (Visit 3, Day 16 ± 1) by a calibrated examiner blinded to treatment allocation.

Microbial Analysis

Salivary samples were obtained from all participants under standardized conditions following a 12-hour overnight abstinence from food and oral hygiene procedures. Samples were transported immediately to the microbiology laboratory and processed within two hours of collection. Serial dilutions of saliva samples were prepared in sterile saline and plated on selective and nonselective culture media appropriate for the target organisms. *Streptococcus *spp. and *Lactobacillus *spp. were cultured on selective agar media under microaerophilic conditions, while gram-negative periodontal pathogens including *Porphyromonas gingivalis*, *Prevotella intermedia*, and *Fusobacterium nucleatum *were cultured using anaerobic incubation conditions. Gram-positive bacterial counts were determined from the combined colony-forming units (CFUs) of *Streptococcus *spp. and *Lactobacillus *spp., whereas gram-negative bacterial counts were determined from the combined CFUs of *P. gingivalis*, *P. intermedia*, and *F. nucleatum*. *Candida albicans *was cultured using fungal selective media.

Plates were incubated at 37°C for 24-72 hours depending on the organism. CFUs were counted and expressed as CFU/mL of saliva. Quantitative microbiological analyses were performed only for samples demonstrating detectable baseline microbial growth for the respective organisms. Consequently, the number of observations for specific microbial endpoints varied between groups depending on baseline culture positivity and organism detection. This approach is consistent with standard microbiological analyses in oral health studies where culture-negative samples cannot be quantitatively analyzed.

Additional Assessments

Clinical oral dryness was assessed at all study visits using the CODS, a validated 10-point objective clinical scoring system that evaluates observable signs of oral dryness, including salivary pooling, mucosal appearance, tongue characteristics, and other clinical features of xerostomia. Breath odor was evaluated using the OSS, a validated sensory assessment based on a 0-5 scale, where 0 indicated no detectable odor, and 5 indicated extremely strong oral malodor. Before each assessment, participants were instructed to refrain from eating, drinking, smoking, and performing oral hygiene procedures for at least 12 hours and to keep their mouths closed for approximately one minute before slowly exhaling through the mouth. Breath odor was assessed by the same trained examiner at a standardized distance of approximately 10 cm under controlled clinical conditions to minimize inter-examiner variability. Tooth shade was assessed using the VITA Classical Shade Guide under standardized clinical lighting conditions. The maxillary central incisors served as the reference teeth for shade comparison, and changes in shade scores from baseline to the EOT visit were recorded by the same calibrated examiner.

Safety assessments included recording demographics (age, gender, race, and ethnicity) and relevant medical and medication history during Visit 1. Physical examinations and vital sign measurements (body temperature, pulse rate, blood pressure, and respiratory rate) were conducted during Visits 1, 2, and 3, with additional assessments performed as needed. Pregnancy tests for female participants of childbearing potential included serum testing at screening (Visit 1) and urine tests on Day 0 (Visit 2) and Day 16 ± 1 (Visit 3). Signs and symptoms of COVID-19 infection were evaluated at each visit. Laboratory investigations during Visits 1 and 3 encompassed hematological parameters (e.g., hemoglobin, leukocytes, and platelet count) and serum glutamate pyruvate transaminase (SGPT) levels, with blood samples collected as per protocol. Protocol-specific tests or clinical evaluations were conducted at the PI’s discretion. Adverse events (AEs) and serious adverse events (SAEs) were recorded from informed consent signing until study completion, regardless of their relationship to treatment. Concomitant medication use was assessed throughout the study duration.

Ethical Considerations

This study was reviewed and approved by the Dixit Hospital Institutional Ethics Committee, Vapi, Gujarat, India (Approval No.: CCS/2023/001; Approval Date: June 22, 2023). The study was conducted in accordance with the ethical principles of the Declaration of Helsinki and applicable institutional guidelines. Written informed consent was obtained from all participants prior to enrollment.

Statistical Methods

Clinical outcomes were analyzed using the full analysis population consisting of all participants who completed the study assessments. Outcomes derived from ordinal scales, including CODS, OSS, and VITA shade scores, were analyzed descriptively and using parametric comparisons for exploratory purposes. These analyses were intended to identify potential trends rather than establish definitive treatment effects. Microbiological analyses were conducted using available culture-positive samples for the respective organisms; therefore, subgroup sizes varied across microbial endpoints.

Statistical analyses were performed using SPSS version 27 (IBM Corp., Armonk, NY). Continuous variables are presented as mean ± standard deviation (SD), whereas categorical variables are presented as frequencies and percentages. Between-group comparisons of continuous outcomes were performed using one-way analysis of variance (ANOVA). Statistical significance was defined as a two-sided p-value < 0.05. Microbiological analyses included only participants with detectable baseline cultures for the respective organisms. Safety outcomes were summarized descriptively.

Clinical efficacy endpoints were analyzed using change-from-baseline values between baseline (Day 0) and EOT (Day 16 ± 1). Between-group comparisons of continuous outcomes were performed using one-way ANOVA when assumptions of normality were satisfied.

Microbiological outcomes were analyzed descriptively and using ANOVA, where appropriate, among participants with detectable baseline cultures.

The analysis population included all participants who completed the study assessments, and analyses were conducted using available data. Given the exploratory nature of several secondary outcomes, adjustments for multiple comparisons were not applied, and findings should therefore be interpreted with caution. Safety outcomes were summarized descriptively using frequency tables.

## Results

Baseline characteristics

A total of 63 participants were randomized, with 21 participants allocated to each treatment group. Baseline demographic and anthropometric characteristics were generally well balanced across the three study groups, with no clinically meaningful differences in age, sex distribution, height, weight, body mass index (BMI), or ethnicity. All participants were of Asian ethnicity, and the overall study population comprised 29 males (46.0%) and 34 females (54.0%). The baseline characteristics of the study population are summarized in Table 2.

**Table 2:** Demographic and other baseline characteristics of the study population

Characteristics	Treatment Group			Overall
	Test (N = 21)	Active comparator (N = 21)	Placebo (N = 21)	N = 63
Sex: Male/Female (n%/n%)	11/10 (52.4%/47.6%)	11/10 (52.4%/47.6%)	7/14 (23.8%/66.7%)	29/34 (42.9%/57.1%)
Age mean (±SD)	33.1 (±8.7)	35.1 (±8.0)	31.8 (±10.7)	33.3 (±9.2)
Height mean (±SD)	161.7 (±8.9)	160.4 (±9.5)	159.5(±10.1)	160.6 (±9.4)
Weight (kg) mean (±SD)	65.5 (±17.0)	67.0 (±12.2)	64.2 (±17.2)	65.6 (±15.4)
BMI (kg/m²) mean (±SD)	24.8 (±5.0)	26.0 (±4.2)	25.1 (±5.9)	25.3 (±5.0)
Ethnicity - Asian n(%)	21 (33.3)	21 (33.3)	21(33.3)	63(100)

Primary efficacy endpoint analysis

All randomized participants completed the clinical assessments (N = 21 per group). However, microbiological analyses included only participants with detectable baseline cultures for the respective organisms, resulting in smaller subgroup sample sizes.

Variability in microbial CFU counts was observed across groups, which is consistent with the heterogeneous distribution of oral microbiota among individuals. A summary of the primary and secondary efficacy outcomes together with the corresponding statistical analyses is presented in Table 3.

**Table 3:** Summary of primary and secondary efficacy outcomes and between-group statistical comparisons

Outcome	Test (Mean ± SD)	Active comparator (Mean ± SD)	Placebo (Mean ± SD)	Statistical test	Test statistic	p-value
Gram-positive bacteria (% reduction)	69.3 ± 39.0	63.5 ± 42.0	51.6 ± 40.0	One-way ANOVA	F(2,36) = 0.69	0.51
Gram-negative bacteria (% reduction)	24.9 ± 3.2	22.1 ± 5.4	11.0 ± 10.1	One-way ANOVA	F(2,16) = 8.01	0.004
Modified gingival index (MGI)	2.7 ± 3.3	−1.4 ± 3.6	−1.1 ± 6.8	One-way ANOVA	F(2,59) = 4.61	0.015
Plaque index (PI)	30.6 ± 10.6	15.5 ± 11.4	2.7 ± 17.0	One-way ANOVA	F(2,59) = 23.02	<0.001
Clinical Oral Dryness Score (CODS)	0.9 ± 0.6	−1.9 ± 0.4	−1.0 ± 0.7	One-way ANOVA	F(2,59) = 124.06	<0.001
Organoleptic Scoring Scale (OSS)	1.52 ± 0.90	0.75 ± 0.50	−0.14 ± 0.60	One-way ANOVA	F(2,59) = 30.37	<0.001
VITA Shade Scale	0.9 ± 1.7	−0.3 ± 1.3	−0.3 ± 0.9	One-way ANOVA	F(2,59) = 5.56	0.011

Effect on CFUs of Gram-Positive Bacteria

**Figure 2:**
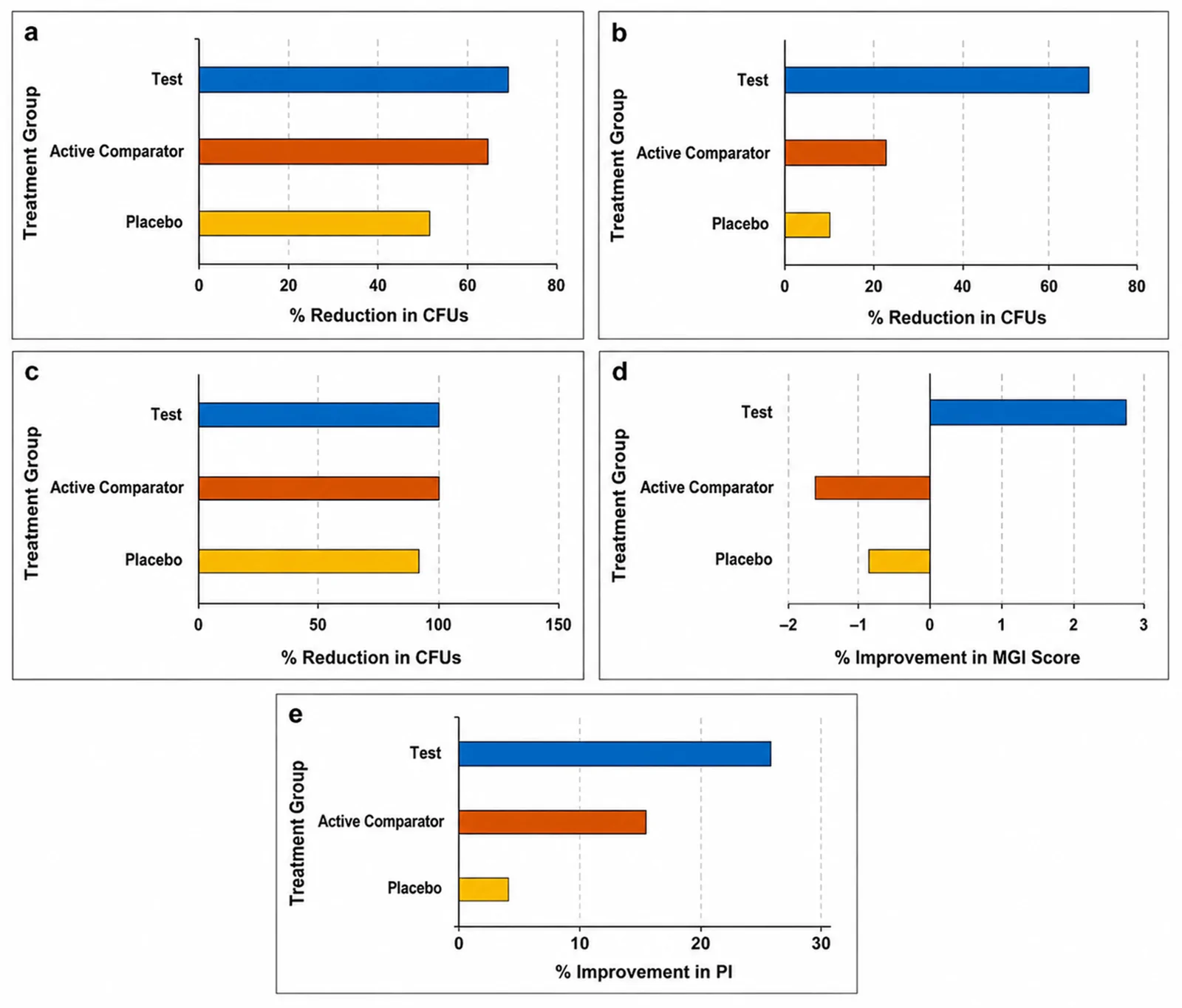
Primary efficacy endpoint analysis (a) Percentage reduction in CFUs of gram-positive bacteria from baseline following treatment with the test oil-pulling oral rinse, active comparator, and placebo. (b) Percentage reduction in CFUs of gram-negative bacteria from baseline across the three study groups. (c) Percentage reduction in *Candida albicans* CFUs from baseline after the intervention. (d) Mean improvement in MGI score from baseline. (e) Mean improvement in PI from baseline. CFUs: Colony-forming units; MGI: Modified gingival index; PI: Plaque index.

At baseline, among participants with detectable cultures for gram-positive organisms, the mean CFUs (in 10⁶ CFUs/mL) were 1.35 (±0.11) in the test group (n = 15), 1.40 (±0.05) in the active comparator group (n = 11), and 1.38 (±0.05) in the placebo group (n = 13). At the EOT, mean CFUs decreased to 0.42 (± 0.53), 0.51 (± 0.59), and 0.66 (± 0.55) for the test, active comparator, and placebo groups, respectively (Figure 2, Panel a). The test group demonstrated a numerically greater mean reduction (69.3 ± 39%), followed by the active comparator (63.5 ± 42%) and placebo (51.6 ± 40%) groups. Between-group comparisons were performed using one-way ANOVA, which showed no statistically significant difference among the three groups (F(2,36) = 0.69, p = 0.51) (Table 3).

Effect on CFUs of Gram-Negative Bacteria

Baseline mean CFUs (in 10⁶ CFUs/mL) of gram-negative bacteria were 1.42 (± 0.04) for the test group (N = 8), 1.36 (± 0.06) for the active comparator group (N = 5), and 1.33 (± 0.16) for the placebo group (N = 6). At EOT, mean CFUs were reduced to 1.06 (± 0.03) for both the test and active comparator groups and 1.18 (± 0.13) for the placebo group. One-way ANOVA demonstrated significant differences among treatment groups. The mean percentage reduction was highest in the test group (24.9 ± 3.2%), followed by the active comparator (22.1 ± 5.4%) and placebo (11.0 ± 10.1%) groups (Figure 2, Panel b). Median reductions were 25.3%, 23.4%, and 10.8%, respectively. The test group demonstrated a 25.2% greater reduction in CFUs than the active comparator group (F(2,16) = 8.01, p = 0.004) (Table 3).

Effect on CFUs of Fungal Microbe

At baseline, the mean CFUs (in 10⁶ CFUs/mL) of *C. albicans* were 1.45 (± 0) in the test group (N = 1), 1.0 (± 0.7) in the active comparator group (N = 3), and not applicable for the placebo group due to an absence of observations. At EOT, both the test and active comparator groups showed complete elimination of *C. albicans *(mean CFUs = 0), resulting in a 100% mean and median reduction from baseline (Figure 2, Panel c). Statistical analysis was not possible due to insufficient data in the placebo group.

Effect on MGI

One-way ANOVA demonstrated significant differences in percentage change in MGI among the three treatment groups. The test group (N = 21) showed a positive mean reduction of 2.7% (± 3.3), whereas the active comparator (N = 20) and placebo (N = 21) groups demonstrated negative mean reductions of -1.4% (± 3.6) and -1.1% (± 6.8), respectively (Figure 2, Panel d). The differences were statistically significant (F(2,59) = 4.61, p = 0.015) (Table 3); however, the magnitude of change in MGI was modest and should be interpreted cautiously in terms of clinical relevance.

Effect on PI

The analysis of percentage reduction in PI showed significant differences among groups. The test group (N = 21) demonstrated the most substantial reduction, with a mean of 30.6% (± 10.6), compared to 15.5% (± 11.4) in the active comparator (N = 20) and 2.7% (± 17.0) in the placebo (N = 21) groups (Figure 2, Panel e). The differences were highly significant (p < 0.001), indicating a robust effect of the test intervention on plaque reduction.

 Secondary efficacy endpoint analysis

A summary of the secondary efficacy outcomes together with the corresponding statistical analyses is presented in Table 4.

**Table 4:** Summary of secondary efficacy outcomes and between-group statistical comparisons

Outcome	Test group (Mean ± SD)	Active comparator (Mean ± SD)	Placebo (Mean ± SD)	Statistical test	Test statistic	p-value
Clinical Oral Dryness Score (CODS)	0.9 ± 0.6	−1.9 ± 0.4	−1.0 ± 0.7	One-way ANOVA	F(2,59) = 124.06	<0.001
Organoleptic Scoring Scale (OSS)	1.52 ± 0.90	0.75 ± 0.50	−0.14 ± 0.60	One-way ANOVA	F(2,59) = 30.37	<0.001
VITA Shade Scale	0.9 ± 1.7	−0.3 ± 1.3	−0.3 ± 0.9	One-way ANOVA	F(2,59) = 5.56	0.011

Effect on CODS From Baseline

**Figure 3:**
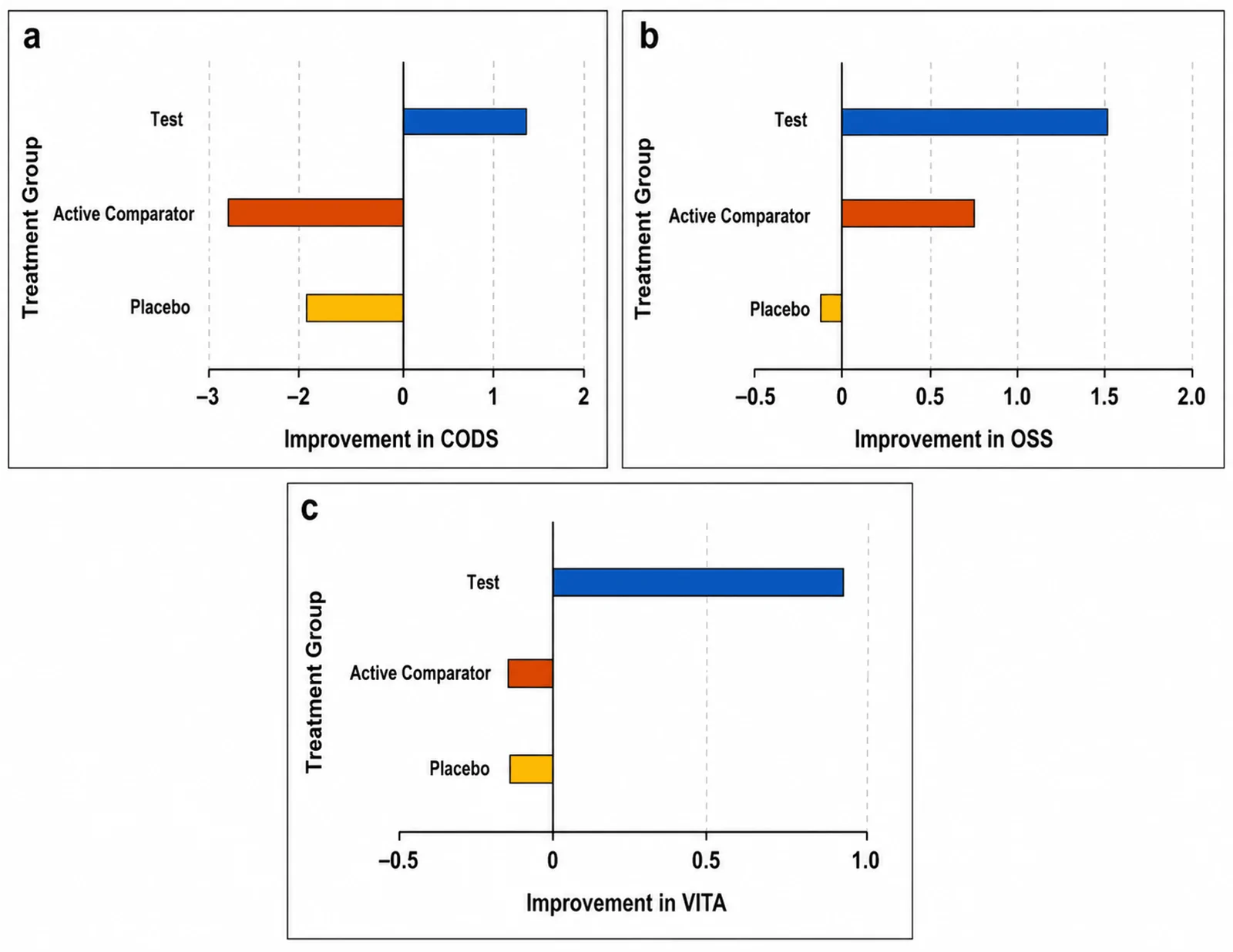
Secondary efficacy endpoint analysis (a) Mean improvement in CODS from baseline following treatment with the test oil-pulling oral rinse, active comparator, and placebo. (b) Mean improvement in OSS score from baseline, reflecting changes in oral malodor. (c) Mean improvement in tooth shade measured using the VITA Classical Shade Guide (VITA) from baseline across the three study groups. CODS: Clinical Oral Dryness Score; OSS: Organoleptic Scoring Scale; VITA: VITA Classical Shade Guide.

The test group demonstrated the greatest improvement in CODS, with a mean improvement of 0.9 ± 0.6, compared with −1.9 ± 0.4 in the active comparator group and −1.0 ± 0.7 in the placebo group (Figure 3, Panel a). Between-group comparisons were performed using one-way ANOVA, which demonstrated a highly significant difference among the three treatment groups (F(2,59) = 124.06, p < 0.001) (Table 4).

Effect on OSS From Baseline

The test group exhibited the greatest improvement in OSS scores (1.52 ± 0.90), followed by the active comparator group (0.75 ± 0.50), whereas the placebo group showed minimal improvement (−0.14 ± 0.60) (Figure 3, Panel b). Between-group comparisons using one-way ANOVA demonstrated a statistically significant difference among the three treatment groups (F(2,59) = 30.37, p < 0.001) (Table 4).

Effect on Teeth Shade on VITA Shade Scale From Baseline

The test group demonstrated the greatest improvement in VITA Shade Scale scores (0.9 ± 1.7), whereas both the active comparator and placebo groups showed a mean change of −0.3 (Figure 3, Panel c). One-way ANOVA demonstrated a statistically significant difference among the three treatment groups (F(2,59) = 5.56, p = 0.011) (Table 4).

Safety Results

Safety outcomes were comparable across treatment arms, with similar exposure and treatment compliance observed. Overall, two mild AEs were reported, one in the test arm and one in the active comparator arm. Both arms experienced a 4.8% incidence of burning oral sensation, categorized under oropharyngeal conditions, while no occurrences were observed in the placebo arm, resulting in an overall incidence of 3.2%. No dose adjustments were required for any IP, and all AEs resolved spontaneously during the study period without the need for additional treatments. Importantly, no serious AEs were reported. Vital signs, physical examinations, and laboratory parameters remained clinically insignificant in all arms. Treatments were well-tolerated, with observed AEs potentially attributable to the study drug or active comparator.

## Discussion

Herbal products have a long-standing history of use in dentistry for combating microorganisms, reducing inflammation, alleviating irritation, and managing pain [12,17,18]. Recent studies have shown promising outcomes with herbal mouthwashes in controlling plaque and gingivitis [19,20]. These mouthwashes are formulated using extracts and essential oils from medicinal plants, incorporating a blend of active components such as catechins, tannins, and sterols [21,22]. The natural compounds in plant-derived substances typically exhibit mild therapeutic effects. Unlike synthetic chemicals, herbal mouthwashes often provide additional anti-inflammatory and antioxidant benefits, which can further enhance gingival health [23]. Various herbal mouthwashes have been developed and evaluated, yet the existing literature presents conflicting findings about their clinical effectiveness in controlling dental plaque and gingival inflammation when compared to placebo or chlorhexidine (CHX) [24-28].

The present study evaluated the comparative efficacy of the novel oil-pulling oral rinse against a marketed alcohol-based anti-gingivitis mouthwash and placebo in improving oral health parameters. The findings suggest potential improvements across microbiological, clinical, and patient-reported measures associated with the test oral rinse. The test product demonstrated numerically greater reductions in gram-positive bacterial counts compared with the comparator and placebo groups. While these differences were not statistically significant, possibly due to sample size limitations, they suggest potential antimicrobial efficacy. This aligns with previous research by Griessl et al., who demonstrated that oil pulling effectively reduces oral bacterial levels and captures representative oral microbiota [29].

For gram-negative bacteria, the test product achieved significantly greater reduction (24.9%) compared to the active comparator (22.1%) and placebo (11.0%) (p = 0.004). This finding is particularly relevant given that gram-negative bacteria are often associated with periodontal diseases. The result extends previous observations by Peng et al., who reported oil pulling's effectiveness in reducing salivary bacterial colonies [30].

The complete elimination of *C. albicans *in both test and active comparator groups supports earlier findings about oil pulling's antifungal properties. A 2006 study by Thorgeirsdóttir et al. examined monocaprin acid’s potential as a denture disinfectant, finding enhanced anti-*Candida* activity upon topical application. Similarly, research conducted in Nigeria demonstrated that 100% coconut oil exhibited antifungal effects against certain *Candida *species, showing comparable efficacy to fluconazole [31]. This corroborates research demonstrating coconut oil’s effectiveness against *C. albicans* [32], although our small sample size for this parameter warrants cautious interpretation.

The improvement in MGI scores in the test group (+2.7%) compared to deterioration in the active comparator (-1.4%) and placebo (-1.1%) groups (p = 0.015) indicates potential anti-inflammatory benefits. This outcome partially contrasts with Peng et al.’s meta-analysis, which found no significant effects on gingival health measurements [30]. However, it aligns with Ripari et al.’s findings of significant improvements in gingival health following oil-pulling therapy [33].

The substantial reduction in PI observed in the test group (30.6%) compared to the active comparator (15.5%) and placebo (2.7%) groups (p < 0.001) represents a notable finding. The plaque reduction observed in the test group falls within the range reported in previous studies evaluating oral hygiene interventions [34].

The test product demonstrated significant advantages in addressing oral dryness, as evidenced by CODS improvements (+0.9) compared to deterioration in other groups (-1.9 and -1.0, p < 0.001). This finding is particularly relevant given the common side effect of oral dryness associated with conventional mouthwashes [7].

The superior improvement in OSS for the test group (1.52) versus active comparator (0.75) and placebo (-0.14) groups (p < 0.001) aligns with previous studies showing oil pulling’s effectiveness against halitosis [35-37]. This suggests that the essential oil blend effectively addresses underlying causes of oral malodor.

The positive change in teeth shade observed in the test group (0.9) compared to slight deterioration in other groups (-0.3) (p = 0.011) partially corresponds with Rajab et al.'s findings [38]. While their study showed stronger whitening effects with hydrogen peroxide-based products, our results suggest that oil-based formulations can provide modest aesthetic benefits without associated sensitivity issues. However, as tooth shade was assessed using a visual shade guide rather than instrumental colorimetric analysis, these findings should be interpreted as exploratory and require confirmation using objective colorimetric methods in future studies.

The favorable safety profile observed, with only mild and transient AEs reported (4.8% incidence of burning sensation in both test and active comparator groups), supports previous literature indicating minimal side effects with oil pulling compared to conventional treatments [39-42]. This advantage becomes particularly relevant when considering the documented side effects of CHX-based products, including staining and taste alterations [43].

The findings suggest that the novel test product, GuruNanda® Oil Pulling Oral Rinse, offers a comprehensive approach to oral care, potentially providing advantages over conventional mouthwashes in terms of broad-spectrum antimicrobial activity, plaque control, gingival health improvement, management of oral dryness, breath freshening, teeth whitening, and minimal side effects. These benefits align with the growing interest in natural oral care alternatives and support the product's potential role as an effective adjunct to routine oral hygiene practices.

Although the test oral rinse demonstrated greater improvements across several clinical and microbiological outcomes than both the active comparator and placebo groups, the findings should be interpreted in the context of the relatively short intervention period and the modest sample size. Improvements observed in the placebo group may reflect enhanced oral hygiene practices associated with study participation (Hawthorne effect), the mechanical cleansing effect common to all rinsing interventions, or natural biological variability. In addition, microbiological analyses were performed only in participants with detectable baseline cultures for the respective organisms, resulting in varying sample sizes across microbial endpoints. Although this approach is consistent with standard microbiological practice, it should be considered when interpreting the microbiological findings. Nevertheless, the consistent improvements observed in the test group across multiple clinical and microbiological parameters suggest a potential adjunctive benefit of the oil-pulling oral rinse.

Study limitations

Study limitations include the relatively short duration and modest sample size. Although the sample size was determined based on expected plaque reduction effects reported in prior mouthwash trials, the study may still be underpowered to detect small differences in certain microbiological outcomes. Therefore, microbiological findings should be interpreted cautiously and confirmed in larger trials.

Additional limitations include the relatively small sample size and the broad age range of the study population, which may have introduced variability in clinical outcomes and limited the generalizability of the findings. Future multicenter studies involving larger cohorts and more homogeneous age groups are warranted to validate these results.

Gingival inflammation was assessed using the MGI rather than bleeding on probing (BOP), which is included in the 2018 American Academy of Periodontology (AAP)/European Federation of Periodontology (EFP) gingivitis classification. Although MGI is a validated and widely used index in clinical trials evaluating oral hygiene products, the absence of BOP measurements limits direct comparison with studies applying the newer periodontal classification framework. Future studies incorporating BOP alongside visual gingival indices would provide a more comprehensive assessment of gingival health.

## Conclusions

This randomized controlled trial suggests that the evaluated oil-pulling oral rinse may serve as a beneficial adjunctive oral hygiene intervention by reducing plaque accumulation and improving selected oral health parameters over a short-term period. Although the test formulation demonstrated greater improvements than both the active comparator and placebo groups for several clinical and microbiological outcomes, the findings should be interpreted in the context of the relatively short intervention period, the modest magnitude of change observed for some outcomes, and the limited sample size.

Consequently, the clinical significance and long-term durability of these effects require further investigation. Larger, multicenter randomized controlled trials with longer follow-up periods are warranted to confirm these findings and establish the long-term efficacy and clinical applicability of the intervention.
